# Community participation and return-to-work outcomes following stroke: a retrospective cohort study of Saudi stroke survivors 1 year after rehabilitation

**DOI:** 10.3389/fneur.2026.1849827

**Published:** 2026-07-29

**Authors:** Mohammad Abu Shaphe, Mohammed M. Alshehri, Ramzi A. Alajam, Ahmed Ghazwani, Ahmad Sahely, Alanoud Huraysi, Basema Temehy, Aafreen, Ashfaque Khan, G. Shankar Ganesh, Abdur Raheem Khan

**Affiliations:** 1 Department of Physical Therapy, College of Nursing and Health Sciences, Jazan University, Jazan, Saudi Arabia; 2 Department of Health Rehabilitation Sciences, Faculty of Applied Medical Sciences, University of Tabuk, Tabuk, Saudi Arabia; 3 Department of Physiotherapy, Integral University, Lucknow, India; 4 Department of Physiotherapy, Composite Regional Centre for Skill Development Rehabilitation and Empowerment of Persons with Disabilities Lucknow, Lucknow, India

**Keywords:** community participation, functional independence, international classification of functioning, disability and health, quality of life, rehabilitation, return to work, stroke

## Abstract

**Background/objective:**

Return to work is an important indicator of successful recovery following stroke; however, vocational reintegration may be influenced by factors extending beyond physical function and disability. Evidence regarding the relationships among community participation, quality of life, functioning, and return-to-work (RTW) outcomes remains limited in Saudi Arabia. The study investigated RTW outcomes, community participation, quality of life, and functional outcomes among stroke survivors 1 year after rehabilitation and examined the associations between community participation and RTW outcomes among stroke survivors.

**Methods:**

A retrospective cohort study was conducted among 150 stroke survivors who completed rehabilitation at a university-affiliated rehabilitation service in Jazan, Saudi Arabia. RTW status at 12 months was recorded as the primary outcome. Community participation, quality of life, disability, cognition, functional independence, activities of daily living, and ICF-based functioning were assessed using validated outcome measures. Correlation analyses and hierarchical regression models were used to examine associations among study variables.

**Results:**

Eighty-five participants (56.6%) returned to paid employment within 12 months of rehabilitation. RTW status was significantly associated with functional independence, community participation, quality of life, cognitive function, and activities of daily living (all *p* < 0.05). Community participation remained independently associated with RTW status after adjustment for demographic and clinical characteristics after adjustment for demographic and clinical characteristics. Participation restrictions persisted despite relatively favourable functional outcomes, indicating a gap between functional recovery and societal reintegration.

**Conclusion:**

Community participation was independently associated with return-to-work outcomes following stroke and may represent an important determinant of long-term societal reintegration. These findings support rehabilitation approaches that extend beyond impairment reduction and emphasise participation, community reintegration, and vocational recovery as key components of long-term stroke rehabilitation.

## Introduction

Stroke remains one of the leading causes of death and long-term disability worldwide, imposing substantial personal, societal, and economic burdens. Improvements in acute stroke management and secondary prevention have increased survival rates, resulting in a growing population of stroke survivors living with persistent physical, cognitive, communication, and psychosocial impairments (1, 2). Consequently, contemporary stroke rehabilitation has shifted from a sole focus on survival and impairment reduction towards the promotion of long-term functioning, participation, and quality of life.

The consequences of stroke extend beyond neurological deficits and frequently affect an individual’s ability to perform daily activities, maintain social relationships, participate in community life, and fulfil occupational responsibilities. These limitations may persist long after formal rehabilitation has ended and can adversely affect independence, psychological well-being, social identity, and financial security. Although traditional rehabilitation outcomes such as mobility, self-care, and functional independence remain important, they do not fully capture how successfully individuals reintegrate into society following stroke (3, 4).

The International Classification of Functioning, Disability and Health (ICF) provides a comprehensive framework for understanding recovery after stroke by conceptualising disability as the interaction between health conditions, body functions and structures, activities, participation, environmental factors, and personal factors (5). Within this framework, participation refers to involvement in life situations and reflects how individuals function within their everyday environments. Participation encompasses engagement in family roles, community activities, recreation, education, religious practices, and employment. Increasingly, participation is recognised as a key outcome of rehabilitation because it represents the extent to which individuals resume meaningful social roles and achieve successful reintegration into society (6, 7).

Community participation is particularly important following stroke because it is strongly associated with psychological well-being, life satisfaction, and quality of life. Participation has therefore become a recommended outcome within contemporary stroke rehabilitation frameworks. Previous studies have demonstrated that participation restrictions frequently persist despite favourable functional recovery, suggesting that improvements in physical function do not necessarily translate into successful societal reintegration (8, 9). These findings have contributed to growing recognition that rehabilitation should extend beyond impairment reduction and address broader outcomes related to participation and community engagement.

Among the various dimensions of participation, return to work (RTW) represents one of the most important outcomes for individuals of working age. Employment provides financial security, social interaction, personal identity, self-esteem, and opportunities for community engagement. Consequently, RTW is frequently regarded as an indicator of successful recovery and societal reintegration following stroke (10). Despite its importance, many stroke survivors experience prolonged unemployment, reduced work capacity, occupational changes, or permanent withdrawal from the workforce. International studies have reported RTW rates ranging from approximately 30 to 60%, reflecting differences in healthcare systems, labour markets, rehabilitation services, and study populations (11, 12).

RTW outcomes are influenced by a wide range of factors including age, educational attainment, stroke severity, functional independence, cognitive function, emotional well-being, workplace demands, and environmental support (13, 14). However, most investigations have focused on isolated predictors rather than examining RTW within a broader biopsychosocial framework. As a result, relatively little is known about how vocational outcomes interact with participation, quality of life, and functioning during long-term recovery.

Quality of life has become another important outcome in stroke rehabilitation research. Health-related quality of life reflects an individual’s perception of physical health, psychological well-being, social relationships, and ability to engage in meaningful activities. Although physical recovery contributes to quality of life, evidence suggests that participation and social reintegration are equally important determinants of long-term well-being (15, 16). Individuals who successfully resume valued social and occupational roles generally report better quality of life than those who remain socially isolated or unemployed. Understanding these interrelationships is therefore important for developing rehabilitation strategies that address the broader consequences of stroke.

The Brief ICF Core Set for Stroke provides a standardised framework for evaluating functioning from a biopsychosocial perspective and enables assessment of domains extending beyond traditional disability measures (17). Previous studies have demonstrated the utility of ICF-based assessments for describing functioning among stroke survivors and identifying factors associated with participation and rehabilitation outcomes (18). Nevertheless, relatively few studies have examined the relationships among ICF-derived functioning profiles, community participation, quality of life, and RTW outcomes within the same population.

These issues are particularly relevant in Saudi Arabia, where stroke represents an increasingly important public health challenge. Rising rates of diabetes mellitus, hypertension, obesity, and physical inactivity have contributed to a growing burden of cerebrovascular disease (19, 20). At the same time, improvements in healthcare have increased survival, resulting in a larger number of individuals living with long-term stroke-related disability. Family support systems, cultural expectations, environmental accessibility, and employment opportunities may further influence recovery and participation outcomes within the Saudi context. Consequently, findings derived from Western populations may not be directly applicable to Saudi stroke survivors (21, 22).

Although previous studies (19–21) in Saudi Arabia have examined functional outcomes and quality of life following stroke, evidence regarding community participation and RTW outcomes remains limited. Further, little is known about how participation, quality of life, functioning, and vocational outcomes interact during long-term recovery. Addressing these gaps may provide valuable information for rehabilitation professionals seeking to promote successful societal reintegration following stroke.

Therefore, this study aimed to investigate RTW outcomes, community participation, quality of life, and functional outcomes among stroke survivors in Jazan, Saudi Arabia, 1 year after rehabilitation. In addition, the study examined the relationships among these outcomes and explored the associations between community participation and RTW outcomes after accounting for demographic and clinical characteristics. Based on previous literature, we hypothesised that greater community participation, better quality of life, and more favourable functional outcomes would be associated with an increased likelihood of returning to work following stroke.

## Methods

This retrospective cohort study examined RTW outcomes, community participation, quality of life, and functional outcomes among stroke survivors who completed rehabilitation at a university-affiliated rehabilitation centre in Jazan, Saudi Arabia. The study was reported in accordance with the Strengthening the Reporting of Observational Studies in Epidemiology (STROBE) Statement (23). Ethical approval was obtained from the Institutional Ethics Committee of Jazan University, Saudi Arabia (REC-44/07/520), and all procedures were conducted in accordance with the Declaration of Helsinki. Data were anonymised before analysis.

Clinical records were reviewed to identify individuals diagnosed with first-ever stroke between June 2021 and November 2022. Stroke diagnosis had been confirmed by specialist physicians using clinical examination and neuroimaging findings. Eligible participants were Saudi nationals aged 18 years or older who had experienced a first-ever ischaemic or haemorrhagic stroke, completed rehabilitation at the university-affiliated rehabilitation service, and were engaged in paid employment before stroke onset. Participants were required to have completed the routine 12-month follow-up assessment after rehabilitation. Individuals with neurological disorders other than stroke, severe communication impairments preventing meaningful assessment, severe cognitive impairment, stroke-like symptoms resulting from non-vascular causes, institutionalisation, or incomplete follow-up data were excluded. Participants who were retired, unemployed, students, or homemakers before stroke onset were also excluded because RTW outcomes could not be meaningfully evaluated in these groups.

**Figure 1:**
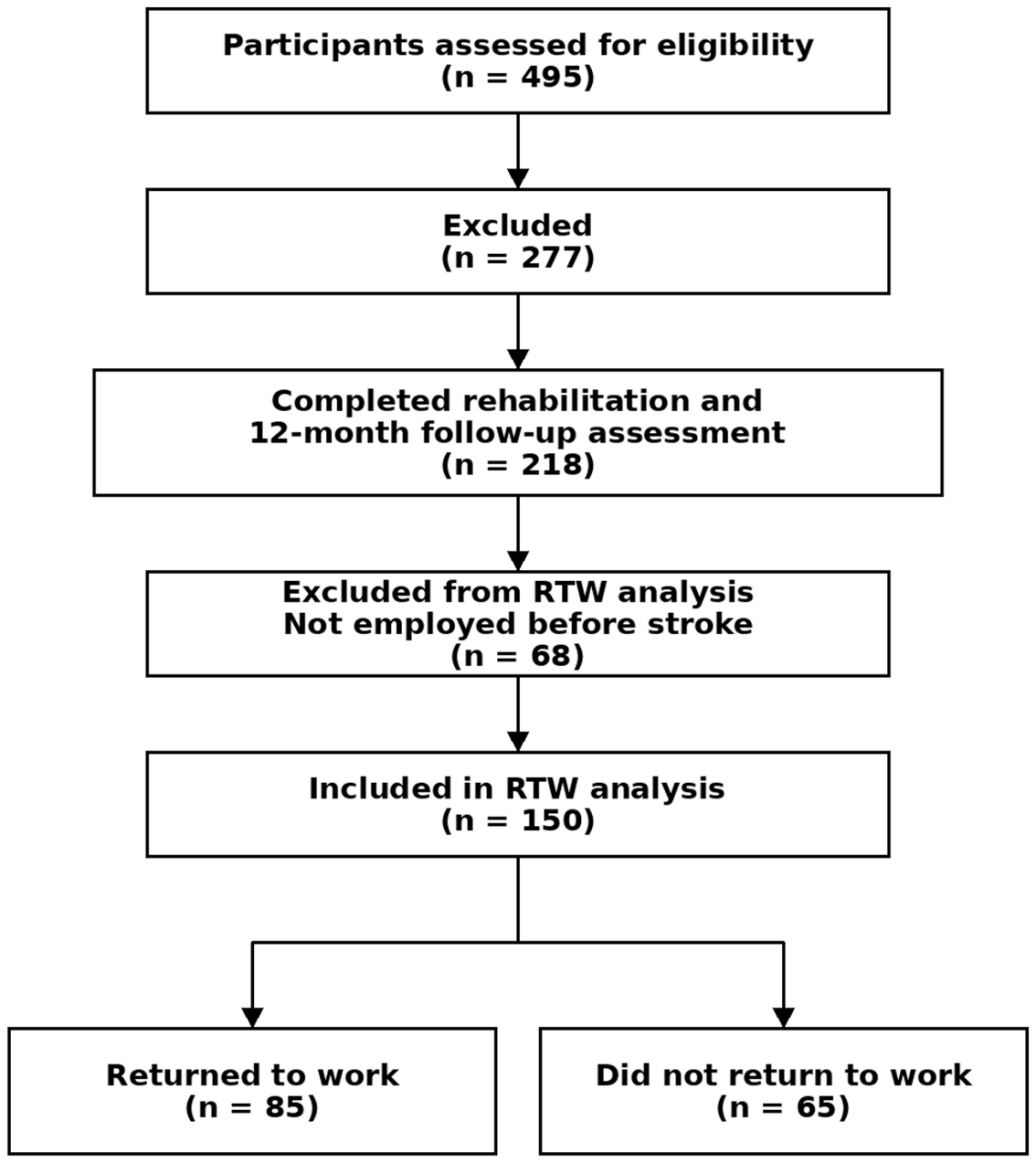
Flow of participants through the study. A total of 495 stroke survivors were assessed for eligibility. Following exclusions and incomplete follow-up, 218 participants completed rehabilitation and the 12-month follow-up assessment. Of these, 68 participants were not employed before stroke and were excluded from the RTW analysis. The final sample comprised 150 participants, including 85 participants who returned to work and 65 participants who did not return to work.

A total of 495 individuals were screened for eligibility. Following application of the inclusion and exclusion criteria, 218 individuals completed rehabilitation and the 12-month follow-up assessment. Of these, 68 participants were not employed prior to stroke and were excluded from RTW analyses. The final study sample comprised 150 participants. The participant selection process is presented in Figure 1.

All participants received multidisciplinary rehabilitation delivered by qualified rehabilitation professionals according to contemporary stroke rehabilitation principles. Interventions were individualised according to clinical presentation, rehabilitation goals, and functional limitations. Programmes typically included physiotherapy, occupational therapy, speech and language therapy where indicated, and psychological support when required. Rehabilitation typically occurred 5 days per week over approximately 12 weeks in duration, although the frequency and content of treatment varied according to individual needs. Prior to discharge, participants and family members received education regarding home-based exercise, mobility, secondary prevention, and maintenance of community activities.

Demographic and clinical information was extracted from medical records and verified through participant interviews when necessary. Variables included age, sex, marital status, educational attainment, family income, residence, family structure, stroke characteristics, duration since stroke, lesion location, side of paralysis, and comorbidity burden. Follow-up assessments were completed approximately 12 months after rehabilitation through face-to-face appointments or structured telephone interviews. All assessments were conducted by physiotherapists experienced in neurological rehabilitation who received standardised training in outcome assessment procedures.

### Sample size estimation

*A priori* sample size estimation was performed using G*Power version 3.1 (24). Because no universally accepted method exists for estimating sample size for hierarchical logistic regression, the calculation was approximated using Cohen’s fixed-model multiple regression approach implemented in G*Power. Assuming a medium effect size (f^2^ = 0.15), *α* = 0.05, power = 90%, and 14 predictors, the minimum required sample size was 112 participants. The final sample of 150 participants exceeded this requirement. In addition, the final model satisfied the commonly recommended minimum of 10 outcome events per predictor variable.

### Outcome measures

RTW status was the primary outcome. RTW was defined as resumption of any form of paid employment at 12 months after stroke, including return to a previous position, a modified role, a new occupation, part-time work, or self-employment.

Community participation was assessed using the Participation Survey/Mobility and Participation Survey (PAR-PRO), which has demonstrated reliability for assessing participation among people with mobility limitations (25). Higher PAR-PRO scores indicate greater community participation and mobility. Health-related quality of life was assessed using the Arabic EQ-5D-3L; Arabic validation studies reported acceptable internal consistency, construct validity, and test–retest reliability, with Cohen’s *κ* ranging from 0.48 to 1.00 in Jordan and 0.53 to 1.00 in Saudi Arabia (26). Global disability was evaluated using the modified Rankin Scale (mRS), which has demonstrated moderate inter-rater reliability, strong test–retest reliability, and established construct and convergent validity in stroke populations (27). Cognitive status was assessed using the Arabic Mini-Mental State Examination (MMSE), which demonstrated intra-rater reliability of 0.89, inter-rater reliability of 0.72, Cronbach’s *α* of 0.71, sensitivity of 80%, specificity of 89.4%, and an area under the curve of 0.92 (28). Functional independence was assessed using the Functional Independence Measure (FIM), which has established reliability and validity in rehabilitation populations (29). Basic and instrumental activities of daily living were assessed using the Barthel Index and Older Americans Resources and Services (OARS) scale, respectively. Functioning from the perspective of the International Classification of Functioning, Disability and Health was evaluated using the Brief ICF Core Set for Stroke (17). Participants were categorised into best, intermediate, and poor quality-of-life groups according to pooled EQ-5D score tertiles. Arabic versions were available for the EQ-5D-3L and MMSE, whereas the remaining instruments were administered using standard clinician-rated procedures that do not require formal language adaptation.

### Statistical analysis

All statistical analyses were performed using IBM SPSS Statistics version 25.0 (IBM Corp., Armonk, NY, USA). Continuous variables are reported as means and standard deviations, whereas categorical variables are presented as frequencies and percentages. Data were screened for missing values, outliers, and violations of statistical assumptions before inferential analyses. Normality of continuous variables was assessed using the Shapiro–Wilk test. Missing data were minimal (<5%) and were handled using complete-case (listwise deletion).

Associations between RTW status and continuous variables were examined using point-biserial correlation coefficients because RTW status was coded as a dichotomous variable. Relationships involving Brief ICF Core Set scores were evaluated using Spearman rank correlation coefficients because ICF qualifier ratings are ordinal in nature.

Hierarchical binary logistic regression analysis was performed to explore the relative associations of demographic, clinical, and participation-related variables with RTW outcomes. Predictor variables were entered in two sequential blocks. Block 1 included demographic and clinical characteristics, whereas Block 2 additionally included community participation (PAR-PRO). This allowed assessment of the incremental increase in Nagelkerke *R*^2^ attributable to community participation beyond demographic and clinical variables (30). Community participation was entered separately in the second block because it represented the primary explanatory variable of interest, whereas the remaining variables were treated as demographic or clinical covariates based on the study hypothesis. Categorical predictors with more than two categories were entered into the regression model using dummy coding, with the lowest-risk or most clinically relevant category serving as the reference group. Binary variables were coded using indicator variables, whereas ordinal variables were entered according to their natural ordering. Statistical significance was established at *p* < 0.05.

## Results

### Participant characteristics

A total of 495 individuals were screened for eligibility during the study period. Following application of the eligibility criteria, 218 stroke survivors completed rehabilitation and the 12-month follow-up assessment. Of these, 68 individuals were not employed before stroke and were therefore excluded from RTW analyses. The final study sample consisted of 150 participants, including 85 who returned to work and 65 who did not return to work at follow-up (Figure 1).

The mean age of participants was 57.75 ± 7.90 years, and the mean duration since stroke was 13.01 ± 0.75 months. Most participants were male (78.7%), married (75.3%), and had experienced an ischaemic stroke (68.0%). More than half had no formal education (59.3%), reported a monthly family income below 20,000 SAR (52.0%), and resided in urban communities (77.3%). Detailed demographic and clinical characteristics are presented in Table 1.

**Table 1:** Demographics and characteristics of study sample.

Variable	*M* (SD)	Range
Age (years), *M* (SD)	57.75 (7.9)	44–69
Stroke duration (months), *M* (SD)	13.01 (0.75)	12–15
	*n* (%)	
Sex		
Male	118	78.6%
Female	32	21.3%
Marital status		
Married	113	75.3%
Widowed/single	37	24.6%
Type of stroke		
Ischemic	102	68.0%
Haemorrhagic	48	32.0%
Education		
No education	89	59.3%
Primary school	17	11.3%
High school	28	18.6%
College	16	10.6%
Employment status (at 12-month follow-up)		
Unemployed	65	43.3%
Employed	85	56.6%
Monthly family income		
< 20,000 SAR	78	52.0%
20,000–29,999 SAR	44	29.3%
>30,000 SAR	28	18.6%
Type of community		
Rural	34	22.6%
Urban	116	77.3%
Family Structure		
Extended family	42	28.0%
Nuclear family	108	72.0%
Number of comorbidities		
0	23	15.3%
1	48	32.0%
2	46	30.6%
3 and above	33	22.0%
Numbers of recurrent strokes		
0	121	80.6%
1	23	15.3%
2 and above	06	4.0%
Side of Paralysis		
Right	90	60.0%
Left	60	40.0%
Localization of lesion		
Frontal lobe	57	38.0%
Parietal lobe	37	24.6%
Temporal lobe	19	12.6%
Occipital lobe	09	6.0%
Brain stem	04	2.6%
Basal ganglia and internal capsule	03	2.0%
Multiple locations	21	14.0%
Setting		
Urban	78	52.0%
Rural	72	48.0%

Participants were recruited from both urban and rural settings. “Type of Community” refers to the participant’s place of residence, whereas “Setting” refers to the location where rehabilitation services were delivered.

### RTW outcomes

At 12 months after stroke, 85 participants (56.6%) had returned to paid employment, whereas 65 (43.3%) had not returned to work (Table 2). RTW status differed significantly according to age, educational attainment, family income, recurrent stroke status, cognitive function, disability severity, functional independence, quality of life, and community participation (all *p* < 0.05). Participants who returned to work had better cognitive performance, lower disability levels, greater functional independence, better quality-of-life, and greater community participation than those who remained unemployed.

**Table 2:** Associations between RTW status and sociodemographic, clinical, and functional characteristics.

Variable	Total *n* (%)	No return to work *n* (%)	Return to work *n* (%)	*p*
Sex				
Female	32 (21.3)	22 (33.8)	10 (11.8)	0.312
Male	118 (78.7)	43 (66.2)	75 (88.2)	
Age				
< 50	37 (24.7)	19 (29.2)	17 (20.0)	< 0.01
50–59	58 (38.7)	23 (35.4)	31 (36.5)	
≥ 60	55 (36.7)	23 (35.4)	37 (43.5)	
Marital status				
Married	113 (75.3)	32 (49.2)	51 (60.0)	0.632
Widowed/single	37 (24.7)	33 (50.8)	34 (40.0)	
Education				
No education	89 (59.3)	31 (47.7)	58 (68.2)	< 0.01
School	17 (11.3)	13 (20.0)	4 (4.7)	
High school	28 (18.7)	17 (26.2)	11 (12.9)	
College and above	16 (10.7)	4 (6.2)	12 (14.1)	
Monthly income				
< 20,000 SAR	78 (52.0)	37 (56.9)	41 (48.2)	0.030
20,000–30,000 SAR	44 (29.3)	15 (23.1)	29 (34.1)	
> 30,000 SAR	28 (18.7)	13 (20.0)	15 (17.6)	
Number of recurrent strokes				
0	121 (80.7)	47 (72.3)	74 (87.1)	< 0.001
1	23 (15.3)	12 (18.5)	11 (12.9)	
≥ 2	6 (4.0)	6 (9.2)	0 (0.0)	
Number of comorbidities				
0	23 (15.3)	11 (16.9)	12 (14.1)	0.232
1	48 (32.0)	17 (26.2)	31 (36.5)	
2	46 (30.7)	16 (24.6)	30 (35.3)	
≥ 3	33 (22.0)	21 (32.3)	12 (14.1)	
MMSE				
< 24	67 (44.7)	61 (93.8)	4 (4.7)	< 0.001
≥ 24	83 (55.3)	4 (6.2)	81 (95.3)	
mRS				
0–1	34 (22.7)	4 (6.2)	30 (35.3)	< 0.001
2	55 (36.7)	12 (18.5)	43 (50.6)	
3–5	61 (40.7)	49 (75.4)	12 (14.1)	
Barthel Index				
< 90	77 (51.3)	58 (89.2)	19 (22.4)	0.021
≥ 90	73 (48.7)	7 (10.8)	66 (77.6)	
OARS-IADL				
< 12	69 (46.0)	48 (73.8)	37 (43.5)	0.090
≥ 12	81 (54.0)	17 (26.2)	48 (56.5)	
FIM				
< 104	89 (59.3)	57 (87.7)	32 (37.6)	< 0.001
≥ 104	61 (40.7)	8 (12.3)	53 (62.4)	
QOL				
< 14	71 (47.3)	6 (9.2)	65 (76.5)	< 0.001
≥ 14	79 (52.7)	59 (90.8)	20 (23.5)	
PAR-PRO				
< 11	85 (56.7)	61 (93.8)	24 (28.2)	< 0.001
≥ 11	65 (43.3)	4 (6.2)	61 (71.8)	

Percentages represent column percentages unless otherwise indicated.

### Quality of life and functional outcomes

The mean pooled EQ-5D-3L score was 15.15 ± 4.57 (Table 3). Difficulties were most frequently reported in the pain/discomfort domain, followed by mobility, self-care, anxiety/depression, and usual activities. Based on pooled scores, 28.6% of participants were classified as having poor quality of life, 48.6% intermediate quality of life, and 22.6% good quality of life.

**Table 3:** Quality-of-life scores based on EQ-5D-3L dimensions.

Level	Mobility	Self care	Usual activities	Pain/discomfort	Anxiety/depression	Pooled scores			*p* value
							*n* (%)	*M* (SD)	
Level 1	51 (34%)	57 (38%)	65 (43.3%)	44 (29.3%)	63 (42%)	All	150 (100%)	15.15 (4.57)	
						Best QOL	34 (22.6%)	10 (2.04)	<0.01
Level 2	60 (40%)	42 (28%)	32 (21.3%)	77 (51.3%)	53 (35.3%)	Intermediate QOL	73 (48.6%)	15 (2.46)	
Level 3	39 (26%)	51 (34%)	53 (35.3%)	29 (19.3%)	34 (22.6%)	Poor QOL	43 (28.6%)	20 (2.16)	

Lower pooled scores indicate better quality of life.

Functional outcomes measured using the modified Rankin Scale indicated that 89 participants (59.3%) achieved favourable outcomes (mRS 0–2), whereas 61 participants (40.7%) continued to experience moderate-to-severe disability (mRS 3–5) 1 year after stroke (Table 4).

**Table 4:** Distribution of Modified Rankin Scale scores at 12-month follow-up.

mRS category at 12 months	*n* (%)	Interpretation
0–1	34 (22.7)	No/minimal dependence
2	55 (36.7)	Slight dependence
3–5	61 (40.7)	Moderate to severe dependence
0–2	89 (59.3)	Favourable functional outcome
3–5	61 (40.7)	Poor functional outcome

### Functioning profile based on the brief ICF Core set for stroke

The distribution of ICF qualifier ratings is presented in Table 5. Most participants reported no impairments in consciousness, orientation, attention, memory, or language functions. In contrast, muscle power impairments remained common, with 78.0% reporting at least mild impairment.

**Table 5:** Distribution of ICF qualifier ratings at 12-month follow-up.

ICF code	No problem *n* (%)	Mild problem *n* (%)	Moderate problem *n* (%)	Severe problem *n* (%)	Complete problem *n* (%)
b110 consciousness function	133 (88.7)	17 (11.3)	0 (0.0)	0 (0.0)	0 (0.0)
b114 orientation function	146 (97.3)	4 (2.7)	0 (0.0)	0 (0.0)	0 (0.0)
b140 attention functions	139 (92.7)	11 (7.3)	0 (0.0)	0 (0.0)	0 (0.0)
b144 memory functions	101 (67.3)	31 (20.7)	14 (9.3)	4 (2.7)	0 (0.0)
b167 mental functions of language	127 (84.7)	6 (4.0)	15 (10.0)	2 (1.3)	0 (0.0)
b730 muscle power functions	33 (22.0)	44 (29.3)	38 (25.3)	34 (22.7)	4 (2.7)
d310 communicating with - receiving spoken messages	119 (79.3)	24 (16.0)	7 (4.7)	0 (0.0)	0 (0.0)
d330 speaking	107 (71.3)	22 (14.7)	21 (14.0)	0 (0.0)	0 (0.0)
d450 walking	73 (48.7)	41 (27.3)	19 (12.7)	17 (11.3)	0 (0.0)
d510 washing oneself	37 (24.7)	67 (44.7)	23 (15.3)	14 (9.3)	9 (6.0)
d530 toileting	109 (72.7)	26 (17.3)	15 (10.0)	0 (0.0)	0 (0.0)
d540 dressing	48 (32.0)	63 (42.0)	23 (15.3)	9 (6.0)	7 (4.7)
d550 eating	84 (56.0)	33 (22.0)	29 (19.3)	4 (2.7)	0 (0.0)

Activity and participation restrictions were also evident. Walking difficulties were reported by 51.3% of participants, while difficulties with washing oneself and dressing were reported by 75.3 and 68.0%, respectively. Difficulties with eating and toileting were less common but remained present in a notable proportion of participants.

### Associations between functioning and clinical outcomes

Correlation analyses are presented in Tables 6, 7.

**Table 6:** Correlation matrix between functioning components of brief ICF core set of stroke and other variables.

Variable	Barthel Index	OARS-IADL	mRS	FIM	EQ-5D-3L	PAR-PRO	Return to work
Brief ICF core set for stroke	−0.59*	−0.43*	0.412*	−0.395*	0.016	−0.167*	0.027

Values are Spearman correlation coefficients (*ρ*). *p < 0.05.

The direction of correlations involving mRS and EQ-5D-3L should be interpreted according to their scoring, where higher scores indicate greater disability or poorer health status.

**Table 7:** Correlation matrix of clinical and participation outcomes.

	Return to work	PAR-PRO	EQ-5D-3L	mRS	FIM	BI	OARS-IADL	MMSE
Return to work	–							
PAR-PRO	0.471*	–						
EQ-5D-3L	−0.438*	0.391*	–					
mRS	−0.346*	−0.401*	−0.283*	–				
FIM	0.642*	0.649*	−0.379*	−0.336*	–			
BI	0.368*	0.157	−0.256*	0.285*	0.438*	–		
OARS-IADL	0.395*	0.362*	−0.050	0.297*	0.478*	0.780*	–	
MMSE	0.402*	0.371*	0.220*	−0.489*	0.578*	0.453*	0.540*	–

Values represent Pearson or point-biserial correlation coefficients as appropriate. *p < 0.05.

The Brief ICF Core Set functioning score showed significant associations with disability and functional independence measures (Table 6). Moderate correlations were observed with the Barthel Index (*ρ* = −0.59), OARS-IADL (*ρ* = −0.43), modified Rankin Scale (*ρ* = 0.41), and Functional Independence Measure (*ρ* = −0.40) (all *p* < 0.05). A weak but significant association was identified between ICF functioning and community participation (*ρ* = −0.17, *p* < 0.05). No significant associations were observed between ICF functioning scores and either quality of life or RTW status.

RTW status demonstrated significant positive correlations with FIM (*r* = 0.642), community participation (PAR-PRO) (*r* = 0.471), cognitive function (MMSE) (*r* = 0.402), instrumental activities of daily living (OARS-IADL) (*r* = 0.395), and basic activities of daily living (Barthel Index) (*r* = 0.368) (all *p* < 0.05). Disability severity measured by the modified Rankin Scale was negatively associated with RTW status (*r* = −0.346, *p* < 0.05). RTW status was also negatively associated with EQ-5D scores (*r* = −0.438, *p* < 0.05), indicating better quality of life among participants who returned to work. Community participation was positively associated with functional independence, cognition, quality of life, and instrumental activities of daily living, while demonstrating a negative association with disability severity (Table 7).

### Exploratory hierarchical binary logistic regression analysis

Regression coefficients, changes in explained variance, and model fit statistics are presented in Table 8.

**Table 8:** Hierarchical binary logistic regression examining factors associated with RTW status.

Predictor	Model 1 B	SE	Wald	OR (95% CI)	*p*-value	Model 2 B	SE	Wald	OR (95% CI)	*p*-value
Age	0.540	0.210	6.63	1.72 (1.14, 2.59)	0.010	0.290	0.211	1.88	1.34 (0.88, 2.02)	0.170
Sex	−0.140	0.275	0.26	0.87 (0.51, 1.49)	0.611	−0.110	0.204	0.29	0.90 (0.60, 1.34)	0.590
Marital status	0.950	0.746	1.62	2.59 (0.60, 11.16)	0.203	0.850	0.771	1.22	2.34 (0.52, 10.59)	0.270
Type of stroke	0.880	0.519	2.87	2.41 (0.87, 6.67)	0.090	0.045	0.025	3.13	1.05 (1.00, 1.10)	0.077
Education	0.740	0.310	5.70	2.10 (1.14, 3.85)	0.017	0.580	0.284	4.18	1.79 (1.02, 3.12)	0.041
Monthly family income	0.880	0.519	2.87	2.41 (0.87, 6.67)	0.090	0.045	0.029	2.48	1.05 (0.99, 1.11)	0.115
Type of community	0.910	0.842	1.17	2.48 (0.48, 12.95)	0.280	0.055	0.031	3.13	1.06 (0.99, 1.12)	0.077
Family structure	0.690	0.694	0.99	1.99 (0.51, 7.77)	0.320	0.069	0.053	1.72	1.07 (0.97, 1.19)	0.190
Number of comorbidities	−0.104	0.046	5.09	0.90 (0.82, 0.99)	0.024	−1.284	0.467	7.55	0.28 (0.11, 0.69)	0.006
Number of recurrent strokes	−0.132	0.073	3.28	0.88 (0.76, 1.01)	0.070	−0.182	0.114	2.55	0.83 (0.67, 1.04)	0.110
Stroke duration (months)	0.800	0.508	2.48	2.23 (0.82, 6.02)	0.115	0.127	0.077	2.71	1.14 (0.98, 1.32)	0.100
Side of paralysis	0.920	0.526	3.06	2.51 (0.90, 7.03)	0.080	0.520	0.282	3.40	1.68 (0.97, 2.92)	0.065
Localization of lesion	0.600	0.437	1.88	1.82 (0.77, 4.29)	0.170	0.600	0.479	1.57	1.82 (0.71, 4.66)	0.210
Community participation	—	—	—	—	—	0.396	0.120	10.83	1.49 (1.17, 1.88)	0.001
	Model 1 Nagelkerke: *R*^2^ = 0.173 (Adjusted 0.162), *p* < 0.001					Model 2 Nagelkerke: *R*^2^ = 0.295 (Adjusted 0.285), Δ*R*^2^ = 0.122, *p* < 0.001				
B, unstandardized regression coefficient; SE, standard error; OR, odds ratio; CI, confidence interval. Model 1 incorporates demographic and baseline clinical features. Model 2 additionally includes community participation (PAR-PRO) entered as the second explanatory block.										

Model stage	Model *χ*^2^	df	Model *p*-value	Nagelkerke *R*^2^	*χ*^2^ Change (Δχ^2^)	Δdf	Change *p*-value
Model 1: baseline demographics & clinical covariates	23.42	13	< 0.01	0.173	23.42	13	< 0.01
Model 2: inclusion of community participation (PAR-PRO)	42.18	14	< 0.001	0.295	18.76	1	0.001

*N* = 150. Model *χ*^2^ represents the omnibus test of model coefficients vs. the intercept-only baseline. *χ*^2^ Change (Δχ^2^) evaluates the statistical significance of adding the community participation parameter stage sequentially.

The first hierarchical regression model, which included demographic and clinical characteristics; Model 1 accounted for 17.3% of the variance in RTW status (Nagelkerke *R*^2^ = 0.173). Within this model, age, educational attainment, and comorbidity burden were significantly associated with RTW outcomes.

After community participation was entered into the model, the Nagelkerke *R*^2^ increased from 0.173 to 0.295 (Δ*R*^2^ = 0.122), indicating that inclusion of community participation substantially improved the explanatory performance of the model (Table 8). Community participation remained independently associated with RTW (OR 1.49, 95% CI 1.17–1.88, *p* = 0.001). Educational attainment and comorbidity burden remained significant in the final model, whereas age was no longer statistically significant.

## Discussion

This study investigated RTW outcomes, community participation, quality of life, and functional outcomes among stroke survivors in Jazan, Saudi Arabia, 1 year after rehabilitation. Three principal findings emerged. First, only 56.6% of participants had returned to paid employment despite completing rehabilitation. Second, substantial participation restrictions persisted even among individuals who achieved relatively favourable functional outcomes. Third, community participation revealed a significant association with RTW status after accounting for demographic and clinical characteristics. Together, these findings suggest that long-term recovery after stroke is influenced by factors extending beyond physical function and disability alone (5, 17). One of the major strengths of the study was the incorporation of multiple validated measures spanning disability, participation, quality of life, cognition, and functioning within a single cohort. Further, the final sample exceeded the minimum sample size estimated *a priori*.

The observed RTW rate is consistent with previous international reports indicating that approximately one-third to two-thirds of stroke survivors resume employment following rehabilitation (11, 12, 14). Variability in reported rates likely reflects differences in healthcare systems, occupational demands, rehabilitation services, labour market conditions, and study populations (13, 31). RTW is widely recognised as an important marker of successful recovery because employment contributes not only to financial security but also to social identity, self-esteem, and community engagement (10). The present findings reinforce the view that vocational reintegration should also be considered a major rehabilitation outcome rather than a secondary consequence of physical recovery (32, 33). The observed association between age and RTW should be interpreted cautiously because it was attenuated after inclusion of community participation and may reflect residual confounding, survivor selection, or characteristics specific to the local employment context rather than an independent effect of chronological age.

Functional independence emerged as an important correlate of RTW outcomes. Participants who returned to work demonstrated better functional independence, lower disability levels, and superior performance in both basic and instrumental activities of daily living. These findings are consistent with previous studies showing that individuals who achieve greater independence in mobility and self-care are more likely to resume employment after stroke (8, 31, 34). Functional independence may facilitate RTW by improving confidence, reducing reliance on caregivers, and enabling individuals to manage occupational demands more effectively (35, 36). However, functional independence alone did not fully explain RTW outcomes, indicating that additional psychosocial, occupational, and environmental factors contribute to successful vocational reintegration (13, 14).

Although comorbidity burden was not significantly associated with RTW status in the univariable group comparisons (Table 2), it emerged as an independent factor in the multivariable logistic regression after adjustment for demographic and clinical covariates. This finding suggests that the relationship between comorbidity burden and RTW may have been influenced by confounding among baseline characteristics, highlighting the importance of multivariable analyses when evaluating factors associated with vocational outcomes following stroke.

One of the most important findings was the persistence of participation restrictions despite relatively favourable functional outcomes. More than half of participants reported difficulties with walking, while substantial proportions continued to experience limitations in dressing and personal care activities. Similar observations have been reported in previous studies, where stroke survivors achieved independence in basic activities of daily living but continued to experience restrictions in social, recreational, and community activities (8, 9, 37). These findings suggest that recovery of functional independence does not necessarily translate into unrestricted participation in everyday life. This distinction highlights the importance of assessing outcomes beyond impairment and disability measures when evaluating long-term recovery (6, 7).

Community participation was independently associated with RTW after adjustment for demographic and clinical characteristics, highlighting its potential importance as a component of long-term rehabilitation. Participation may reflect an individual’s confidence, social connectedness, environmental accessibility, community mobility, and engagement in meaningful activities (6, 7, 9). Individuals who actively participate in community life may have greater opportunities to rebuild social networks, develop self-efficacy, and access vocational opportunities (19, 38). Conversely, restricted participation may contribute to social isolation and reduced opportunities for occupational reintegration (35, 37).

These findings are consistent with the International Classification of Functioning, Disability and Health, which conceptualises disability as the result of interactions among health conditions, body functions and structures, activities, participation, environmental factors, and personal factors (5). Participation restrictions frequently arise from environmental and social barriers rather than impairments alone (6, 7). Limited transportation, inaccessible workplaces, inadequate vocational support, and societal attitudes towards disability may restrict opportunities for employment and community engagement (32, 39). Consequently, interventions targeting participation may provide benefits that extend beyond improvements in physical function (33, 38).

Quality of life was also associated with RTW status and community participation. Participants reporting higher quality of life were more likely to have resumed employment and to engage in community activities. Similar associations have been reported in previous stroke studies, where quality of life was strongly related to participation, social integration, and vocational outcomes (15, 16, 36). Quality of life following stroke reflects multiple dimensions of recovery, including physical health, emotional well-being, social functioning, and satisfaction with daily life (40). The observed associations likely reflect reciprocal relationships among these domains. Participation in meaningful activities and employment may facilitate quality of life through enhanced social integration and financial security, while individuals with higher quality of life may be more motivated to pursue occupational and community roles (15, 16).

Cognitive function demonstrated significant relationships with RTW outcomes, community participation, and functional independence. Cognitive impairments are common after stroke and may affect attention, memory, executive functioning, and problem-solving abilities (41). These deficits can limit an individual’s ability to manage workplace demands, navigate community environments, and perform complex daily activities. The present findings support previous evidence identifying cognitive function as an important factor associated with successful vocational reintegration after stroke (11, 14, 31) and highlight the need for comprehensive cognitive assessment during rehabilitation.

Interestingly, the Brief ICF Core Set functioning score was significantly associated with disability and functional independence but not with quality of life or RTW status. This finding may reflect the fact that the Brief ICF Core Set primarily captures impairments and activity limitations, whereas RTW and quality of life are broader outcomes influenced by environmental, occupational, psychological, and social factors (17, 42). The results therefore reinforce the importance of complementing impairment-based assessments with participation-specific measures when evaluating long-term recovery (6, 18).

The Saudi Arabian context may provide additional insights into these findings. Stroke represents an increasing rehabilitation concern in Saudi Arabia, with growing recognition of long-term disability and rehabilitation needs (19, 20). Family support networks may influence both participation and vocational outcomes by providing practical and emotional assistance during recovery (21, 43). At the same time, cultural expectations regarding caregiving and employment may shape decisions regarding RTW. Educational attainment was significantly associated with RTW outcomes. Individuals with higher levels of education may have greater access to less physically demanding occupations and increased opportunities for workplace accommodation, thereby facilitating vocational reintegration (13, 14). These contextual factors may partly explain differences between the present findings and those reported in Western populations. However, because of the observational design, these findings should not be interpreted as evidence of mediation.

The findings have important implications for rehabilitation practice. Traditional rehabilitation programmes often prioritise restoration of mobility, self-care, and functional independence (35, 41). While these outcomes remain essential, the present study suggests that participation and vocational outcomes should also be considered core rehabilitation goals (32, 38). Incorporating community-based rehabilitation, participation-focused interventions, vocational counselling, workplace assessments, and supported employment programmes may help bridge the gap between functional recovery and successful societal reintegration (33, 39). Rehabilitation professionals may consider routinely assessing participation restrictions and employment goals alongside conventional measures of disability and functional independence.

Several limitations should be considered when interpreting these findings. The retrospective design limits causal inference and prevents determination of temporal relationships among participation, quality of life, and RTW outcomes. The regression analyses were exploratory in nature and should be interpreted as examining associations among study variables rather than establishing causal or predictive relationships. Future prospective studies should externally validate these findings using prespecified multivariable logistic regression models in independent cohorts. Community participation and RTW status were assessed at the same follow-up time point; therefore, reciprocal relationships between these variables cannot be excluded. The study was conducted within a single region of Saudi Arabia and included only individuals employed before stroke, which may limit generalisability. Information regarding acute stroke severity, workplace accommodations, depression, fatigue, and occupational characteristics was unavailable and could not be incorporated into the analyses. In addition, RTW status was assessed as a dichotomous outcome and did not capture employment quality, job retention, productivity, or sustainability of work participation. Residual confounding from unmeasured occupational, psychosocial, and workplace-related factors cannot be excluded. Though missing data were minimal, complete-case analysis may have introduced a small degree of selection bias. Future prospective longitudinal studies should incorporate these variables to provide a more comprehensive understanding of vocational recovery after stroke.

## Conclusion

More than half of stroke survivors in this cohort returned to paid employment within 1 year of rehabilitation; however, substantial participation restrictions persisted despite relatively favourable functional outcomes. Community participation demonstrated a significant association with RTW status after accounting for demographic and clinical factors, highlighting its importance as a key component of long-term recovery. These findings support continued emphasis on a broader biopsychosocial approach that prioritises participation, community reintegration, and vocational recovery. Rehabilitation strategies that address participation restrictions and employment-related barriers may improve long-term outcomes and facilitate successful societal reintegration following stroke.

## Data Availability

The raw data supporting the conclusions of this article will be made available by the authors, without undue reservation.
